# Interplay between Inflammation and Cellular Stress Triggered by *Flaviviridae* Viruses

**DOI:** 10.3389/fmicb.2016.01233

**Published:** 2016-08-25

**Authors:** Ana L. C. Valadão, Renato S. Aguiar, Luciana B. de Arruda

**Affiliations:** ^1^Departamento de Genética, Instituto de Biologia, Universidade Federal do Rio de JaneiroRio de Janeiro, Brazil; ^2^Departamento de Virologia, Instituto de Microbiologia Paulo de Góes, Universidade Federal do Rio de JaneiroRio de Janeiro, Brazil

**Keywords:** flavivirus, innate immune response, ER stress, reactive oxygen species, stress granules

## Abstract

The *Flaviviridae* family comprises several human pathogens, including Dengue, Zika, Yellow Fever, West Nile, Japanese Encephalitis viruses, and Hepatitis C Virus. Those are enveloped, single-stranded positive sense RNA viruses, which replicate mostly in intracellular compartments associated to endoplasmic reticulum (ER) and Golgi complex. Virus replication results in abundant viral RNAs and proteins, which are recognized by cellular mechanisms evolved to prevent virus infection, resulting in inflammation and stress responses. Virus RNA molecules are sensed by Toll-like receptors (TLRs), RIG-I-like receptors (RIG-I and MDA5) and RNA-dependent protein kinases (PKR), inducing the production of inflammatory mediators and interferons. Simultaneously, the synthesis of virus RNA and proteins are distinguished in different compartments such as mitochondria, ER and cytoplasmic granules, triggering intracellular stress pathways, including oxidative stress, unfolded protein response pathway, and stress granules assembly. Here, we review the new findings that connect the inflammatory pathways to cellular stress sensors and the strategies of *Flaviviridae* members to counteract these cellular mechanisms and escape immune response.

## Flavivirus Replication

The *Flaviviridae* family includes several important human pathogens such as Dengue Virus (DENV), Zika Virus (ZIKV), Yellow Fever Virus (YFV), West Nile Virus (WNV), Japanese Encephalitis Virus (JEV), St. Louis Encephalitis Virus (SLE), and Hepatitis C Virus (HCV). In this review, we will describe the inflammation and stress responses triggered by members of flavivirus and hepacivirus genus only. Briefly, the first interaction of Flavivirus with its host cell occurs via several putative receptors (Mukhopadhyay et al., 2005). They play an important role in capturing and concentrating infectious virions, leading to a cascade of events that culminates in the virus and cell membranes fusion. Most Flaviviruses are internalized into the cell by clathrin-mediated endocytosis (Pierson and Kielian, 2013). A low pH-triggered conformational change of envelope (E) proteins in endosomes leads to viral and cellular membranes fusion and virus uncoating. Capsid is released into the cell cytoplasm, where it dissociates and releases RNA viral genome. Flavivirus genome is a single strand positive sense RNA molecule, with a single open reading frame (ORF), which is then translated into one large polyprotein (Iglesias et al., 2009). This polyprotein is targeted to the endoplasmic reticulum (ER), where it is processed by virus and host’s encoded proteases to form the structural proteins (capsid protein C and envelope protein E) and non-structural proteins (NS), which participate in replication, polyprotein processing and virion assembly. Usually, Flavivirus replication takes place at a membranous web associated with the ER (Pierson and Kielian, 2013). Formation of the membranous web is mainly induced by NS viral proteins, and replication is catalyzed by an RNA-dependent RNA polymerase (usually named NS5), via a negative sense RNA intermediate (Mottola et al., 2002; Gillespie et al., 2010). Once capsid proteins coat the genomes copies, immature virions, containing surface E proteins, bud into ER lumen and are transported through the *trans*-Golgi network (TGN). At the TGN, they undergo further glycan modifications and structural cleavage, becoming mature infectious virions, which are transported out of the cell by exocytosis (Mukhopadhyay et al., 2005).

Production of viral progeny interferes with different aspects of cellular metabolism; therefore, viral infection may represent a stress condition to the host cell (Fernandez-Garcia et al., 2009). In order to adapt to the stress, the cells react by transiently inhibiting protein synthesis and restricting the consumption of nutrients and energy, aiming to enhance cell survival and restore homeostasis (Ruggieri et al., 2012; Onomoto et al., 2014). Stress responses triggered by virus infection may occur at multiple levels and include the ER associated stress, mitochondria stress (oxidative stress) and cytoplasmic stress [stress granules (SGs)] (Buchan and Parker, 2009; Montero and Trujillo-Alonso, 2011; Valiente-Echeverría et al., 2012; Zhang and Wang, 2012; Reshi et al., 2014; Gullberg et al., 2015).

All these responses may be triggered by host cell sensing of incoming virus macromolecules, including genome RNA, double-stranded RNA intermediates, and proteins. Virus sensing is achieved by pattern recognition receptors (PRRs), which recruit adaptor molecules and stimulate transcription factors, leading to the expression of type I interferons (IFN) and proinflammatory cytokines (Chang et al., 2006; Kato et al., 2006; Boo and Yang, 2010; Muñoz-Jordán and Fredericksen, 2010). Those, in turn, may regulate the expression and activation of PRRs themselves and of other mediators, which are involved in the control of translation, mitochondrial function, and cell death or survival, contributing to inflammation and stress. Viruses are, then, confronted with the consequences of cell stress and emerging evidences suggest that they not only interfere with the interferon system, but also manipulate the programs of cell-induced stress to promote viral replication (Garaigorta and Chisari, 2009; Aguirre et al., 2012; Green et al., 2014).

Notably, prolonged stress may result in cell death. Indeed, activation of PRR and IFN receptors (IFNR) are usually associated to stimulation of different cell death pathways (Li et al., 2004; Fulda et al., 2010). In turn, intracellular mediators activated by stress responses or released after tissue damage may also stimulate PRRs and adaptor molecules, amplifying the inflammatory response. The overall inflammation and stress are important for limiting virus replication and dissemination and for tissue repair, but may be harmful to the host if an exacerbated, uncontrolled response is triggered (Inohara and Nuñez, 2003).

Recent findings have been demonstrating an intimate interaction between RNA immune sensors and cell stress pathways, which may be central for the success of virus replication controlling (Smit et al., 2011; Perera-Lecoin et al., 2013). This review will focus on the cellular stress responses triggered by flavivirus and HCV replication, addressing the connection between those responses with virus sensing and inflammation, and discussing the virus strategies to counteract these pathways.

## Flavivirus Sensing Complexes

Virus RNA may be sensed by RIG-I-like receptors (RLR) or toll-like receptors members (TLR), depending on RNA structure, cellular location, and infected cell type. Monocytes, macrophages and non-immune cells, such as endothelial cells, epithelial cells and hepatocytes usually sense RNA virus through RIG-I and/or TLR3; whereas TLR7 is highly expressed and is the major RNA sensor in plasmacytoid dendritic cells (pDC; Sun P. et al., 2009; Tsai et al., 2009; Nasirudeen et al., 2011; Qin et al., 2011; da Conceição et al., 2013). Activation of either RLRs or TLRs may promote the secretion of interferons and proinflammatory cytokines. The former induces autocrine and paracrine stress responses, such as inhibition of protein synthesis, RNA editing and, potentially, cell death, contributing to control viral replication and dissemination. Proinflammatory cytokines and chemokines, such as IL-6, IL-8, TNF-α and Rantes, recruit and activate other cell types to the infected tissue, amplifying inflammation. These mediators may also contribute to tissue lesion due to activation of cell death pathways, and induction of oxidative stress, among other mechanisms, which will be discussed later (He et al., 2011; O’Leary et al., 2012; Lucas and Maes, 2013). All these effects may contribute to the control of virus replication, but also to enhancement of inflammatory response and disease severity.

Virus components and cellular metabolites generated upon virus replication may also stimulate inflammasome complexes, leading to the secretion of inflammatory IL-1β and IL-18 and, eventually, to cell death (Poeck et al., 2010; Negash et al., 2013; Chen et al., 2014).

### Cytoplasmic RNA Sensing

**FIGURE 1:**
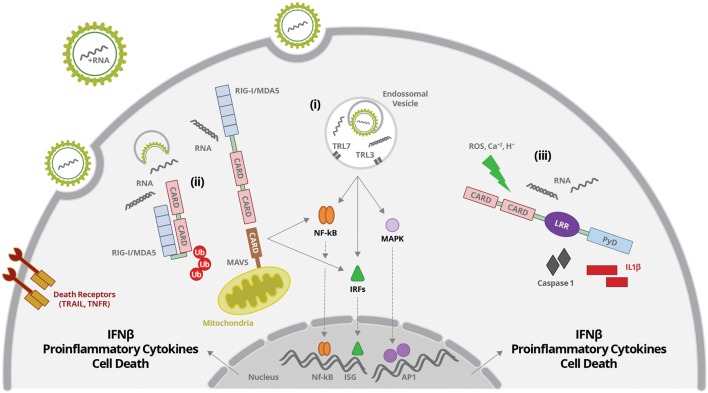
**Flavivirus entry and uncoating is followed by virus RNA sensing by: (i) TLR3 and 7 present in endosomal vesicles; (ii) RIG-I or MDA5 at the cytoplasm; (iii) NOD-like receptors (NLR) at the cytoplasm. (i) ssRNA or dsRNA are sensed by TLR7 or TR3, respectively.** TLR activation leads to activation of IRFs, NF-κB, and MAPK and their translocation to the nucleus, inducing the production of interferons, proinflammatory cytokines and cell death. (ii) dsRNA sensing by RIG-I induces a conformational change of CTD, allowing exposition of CARD. CARD domain is then targeted by the ubiquitin ligases, which promotes its polyubiquitination and MAVS recruitment. RIG-I/MAVS activation promotes activation of IRFs, NF-κB, and MAPK and their translocation to the nucleus, inducing the production of interferons, proinflammatory cytokines and cell death. (iii) RNA sensing together with other stress signals (ROS, increased Ca^2+^ and/or H^+^) activates inflammasome complexes, inducing caspases 1/11 activation and IL-1β and IL-18 secretion.

Virus RNA present in the cytoplasm may be sensed by RLR, including RIG-I and MDA5. These are cytoplasmic helicases, composed by a RNA-binding domain at the C-terminal region (CTD), associated to a central DExD/H helicase domain with an ATP-binding motif, and a caspase recruitment domain (CARD), located at the N terminus (Kowalinski et al., 2011) (**Figure 1**). RLRs recognize, by their RNA-binding domain, signatures present in several RNA virus. dsRNA bearing an uncapped 5′triphosphate end (5′ppp) with a minimum of 20 nt length was showed to be essential for optimal RIG-I sensing, whereas long dsRNA, lacking triphosphate end are preferentially recognized by MDA5 (Kato et al., 2006). Once the receptor is activated, CARD domain associates to the adaptor molecule MAVS (or IPS, VISA, or Cardif), which is located at the outer membrane of mitochondria (Gack et al., 2007) (**Figure 1**). In steady state conditions, CARD domain is masked by CTD, avoiding intrinsic activation of the receptor. The binding of viral RNA signatures leads to an ATP-dependent conformational change of CTD, allowing exposition of CARD (Kowalinski et al., 2011) (**Figure 1**). CARD domain is then targeted by the ubiquitin ligases tripartite motif protein 25 (TRIM25), which promotes the polyubiquitination of lysine-63 (K63), RIG-I oligomerization, and MAVS recruitment, being essential to RIG-I-induced antiviral responses (Gack et al., 2007; Jiang et al., 2012) (**Figure 1**). In addition, ubiquitination of CTD by the ring finger proteins 135 (RNF135 or Riplet), which are upregulated upon virus infection, also facilitates RIG-I–mediated viral recognition (Oshiumi et al., 2009, 2010).

RIG-I-MAVS interaction may then form complexes with other adaptor proteins including: STING (stimulator of interferon genes); ERIS (ER interferon stimulator); TRADD (TNFR-associated death domain); FADD (Fas-associated death domain protein); RIP1 (receptor-interacting protein 1); TRAF2, 3, 6 (TNFR-associated factors 2, 3, and 6) and caspases (Takahashi et al., 2006; Ishikawa and Barber, 2008; Yoshida et al., 2008; Zhong et al., 2008; Sun W. et al., 2009; Rajput et al., 2011). After full activation of the receptor, MAVS recruitment, together with the formed complexes activates TBK and IKK, triggering the stimulation of IRF3 and NF-κB. Activated IRF and NF-κB transcription factors are translocated to the nucleus, where they induce the expression of interferons and proinflammatory cytokines, respectively (**Figure 1**).

Activation of RIG-I, MDA5, MAVS, STING, IRF3, and NF-κB had been reported to mediate sensing of dsRNA intermediates during Flavivirus replication. Those pathways were, then, associated to regulation of IFN production and IFN-mediated responses, as well as to cellular stress or cell death/survival. Depending on the virus and the infected cell type, RNA virus can be distinguished by RIG-I or MDA5, or both receptors may be synergistically stimulated (Kato et al., 2006; Nasirudeen et al., 2011).

Both RIG-I and MDA5 were shown to be up regulated and involved in IFN-β induction upon DENV and WNV infection in hepatocytes and endothelial cells (Loo et al., 2008; Nasirudeen et al., 2011; da Conceição et al., 2013). RIG-I, MDA5 and MAVS were, then, associated to increased secretion of inflammatory mediators, which have been also observed in patients’ plasma. Unexpectedly, RLR silencing did not affect viral replication, indicating that those molecules might be more associated to inflammation than to viral replication.

Zika Virus infection of human fibroblasts also resulted in upregulation of RIG-I and MDA5, what might be associated to the observed production of IFN-α and IFN-β, increased expression of IRF7 and upregulation of IFN stimulated genes (ISG), such as OAS and ISG15 (Hamel et al., 2015).

On the other hand, JEV and HCV are recognized only by RIG-I (Sumpter R. et al., 2005; Kato et al., 2006). The polyuridine motif of HCV 3′ untranslated region (UTR) genome region and its replication intermediates are the PAMP substrates of RIG-I, that activates IRF3, thereby inducing the expression of IFN-α/β and antiviral/interferon-stimulated genes that limit infection (Sumpter R. et al., 2005; Saito et al., 2008). During HCV infection, RIG-I was also associated to the induction of cellular apoptosis through the TRAIL pathway and the death receptors DR4 and DR5 (Eksioglu et al., 2011) (**Figure 1**). Activation of RIG-I-MAVS/STING pathways during JEV infection in neurons was associated to increased production of interferon, proinflammatory cytokines and reduction of intracellular levels of virus (Nazmi et al., 2011, 2012). On the other hand, it was demonstrated that JEV infection induced the expression of microRNAs, such as miRNA15b, which negatively regulates RIG-I signaling, contributing to virus escape from innate immune response (Zhu et al., 2015).

### Vesicular RNA Sensing

Flavivirus genome may also be sensed by TLR, located at endosomal vesicles (Nazmi et al., 2014) (**Figure 1**). TLRs are composed by leucine-rich repeats (LRRs) luminal domain, which is responsible for PAMP recognition; a transmembrane domain; and the Toll/IL-1 receptor domain (TIR), which faces the cytoplasm and associates to downstream signaling molecules (Kawai and Akira, 2010). TLRs may signal through two different pathways: one dependent on the recruitment of the adaptor molecule MyD88, and other involving the adaptor TRIF, which is independent on MyD88. Immune response against Flaviviruses and HCV involves TLR3 and TLR7 activation, which recognize dsRNA and ssRNA containing uridine rich motifs, respectively (**Figure 1**).

After TLR7 engagement, MyD88 is recruited and forms a complex with proteins IRAK1 and IRAK4 (interleukin-1 receptor-associated kinases 1 and 4), TRAF3, and TRAF6 (TNF receptor-associated factors 3 and 6). These complexes stimulate MAPK, IKK and TBK, which will activate and promote the nuclear translocation of AP1, NF-κB, IRFs 3 and 7 transcription factors, inducing the expression of type I interferons, pro-inflammatory cytokines and chemokines (Hemmi et al., 2002; Lin et al., 2010; von Bernuth et al., 2012; Zhou et al., 2013) (**Figure 1**).

Toll-like receptor3 recruits the adaptor molecule TRIF, which also interacts with TRAF3 and 6, activates TBK1 and IKK, stimulating NF-κB and IRF3, and cytokine secretion. TRAF6 also interacts with RIP, which stimulates TAK-1complex, promoting the activation of NF-κB and MAPK (Hemmi et al., 2002; Kawasaki and Kawai, 2014). Activation of TLR3 or TLR7, therefore, promotes the secretion of interferons and proinflammatory cytokines. Also, endosomal TLR may sense self-RNAs released by damaged cells, indicating another cross talk pathway between stress, lesion and innate immune response (Takemura et al., 2014).

Dengue Virus RNA is recognized by TLR3 in monocytes (Tsai et al., 2009; Nasirudeen et al., 2011). Also, TLR3 may synergize with RIG-I and MDA5 for the induction of interferons after DENV infection of hepatocytes (Nasirudeen et al., 2011). Interestingly, DENV infected cells may be sensed by pDCs, in a pathway involving cell-to-cell contact and RNA-dependent TLR7 activation, promoting the production of IFNα, inducing, therefore, an antiviral state (Décembre et al., 2014).

Upregulation of TLR3, but not TLR7 was also observed in human fibroblasts infected by ZIKV (Hamel et al., 2015). In fact, both cytoplasmic (RIG-I and MDA5) and vesicular (TLR3) PRRs were upregulated and might synergistically account for the enhanced expression of antiviral response stimulated after ZIKV infection, however, the specific role of each receptor was not investigated yet (Hamel et al., 2015).

Toll-like receptor-mediated protection was also observed during WNV infection in neurons, although this effect had not been so evident upon infection of other cell types (Daffis et al., 2008). In addition, in an *in vivo* experimental model, TLR3 showed to be essential for WNV penetration in the brain and for the induced inflammatory response (Wang et al., 2004). On the other hand, MyD88-deficient mice presented increased susceptibility to WNV infection, indicating the participation of other TLR in WNV protection and pathogenesis (Szretter et al., 2010). Regarding HCV, TLR3 was strongly involved in the induction of IFNs, which showed to be important to keep low levels of viral replication (Eksioglu et al., 2011).

## Type I Interferons and Induction of Antiviral State

A major virus-induced response upon virus sensing in a range of different cell types is the production of type I IFN. During flavivirus infection, IFN production is stimulated by RIG-I, MDA5, TLR3, and TLR7 triggering a variety of cell responses affecting cellular physiology and virus replication (Muñoz-Jordán and Fredericksen, 2010). Increased IFN production induced by activation of PRRs also contributes to the amplification of the response, by increasing the expression of PRRs themselves and subsequent signal transduction.

The essential role of type I IFNs for the control of Flaviviruses replication is clearly evidenced in experimental mouse models, which do not express IFN receptors or IFN-related signaling molecules, such as STAT. Although adult wild type mice are not susceptible or do not develop classical disease when inoculated with most flaviviruses, infection of A129 mice, lacking type I IFN receptor, result in systemic and fatal infection induced by ZIKV and YFV (Meier et al., 2009; Rossi et al., 2016). Similarly, DENV infection of AG129 mice, which lacks both type I and type II IFN receptors, or infection of STAT2-deficient mice, lead to fatal infection, associated to viral replication in multiple organs, and vascular alterations, including hemorrhage (Williams et al., 2009; Ashour et al., 2010). These data indicate that both innate and adaptive responses are crucial for dengue protection.

Type I IFN binding to IFN receptors (IFNAR) expressed at the surface of the infected or bystander cells promote the expression of a number of ISGs, including PKR, OAS/RNAse L, IFITS, and others, which then modulates protein synthesis, RNA degradation, autophagy and apoptosis (Li et al., 2004; Chakrabarti et al., 2012).

One of the first described antiviral element up-regulated by interferon was the double-stranded RNA-dependent protein kinase (PKR; Xu and Williams, 1998). When activated, it phosphorylates the guanine nucleotide exchange factor eIF2B from recycling eIF2 to its active GTP-bound form, the alpha-subunit of translation initiation factor eIF2-α (eIF2-alpha; Aguirre et al., 2012). This leads to the shutoff of protein synthesis and, thereby, inhibition of viral replication. PKR is activated by binding to dsRNA, and could be, therefore, classified as a RNA sensor (Onomoto et al., 2012). However, PKR is more accurately characterized as a critical sensor of cell stress and virus infection, which activates stress responses and inflammation through dsRNA recognition domain (Wu and Kaufman, 1997; Balachandran et al., 1998). The function of PKR in flavivirus-mediated stress responses will be further detailed in the cytoplasmic stress section.

Other ISGs also indirectly modulate the immune response, such as RNAse L, that activates RIG-I/MAVS pathways, leading to increased IFN-β production triggered by HCV RNA (Malathi et al., 2010). However, the effect of RNAse L in IFN production may vary depending on the virus and the cell type, given that different cell types express different isoforms and levels of OAS genes (Banerjee et al., 2014). Therefore, increased IFN production is one of the key elements for an antiviral response, by amplifying the inflammatory response, by regulating cellular metabolism and consequently affecting virus production. The function of several others ISGs are beyond the scope of this review and we will discuss here how viral RNA and protein sensing and IFN signaling can be associated to cellular stress.

## Cellular Stress Pathways

### ER Stress Response [Unfolded Protein Response (UPR) Pathway] and Inflammation

Endoplasmic reticulum is the major site where secreted and transmembrane proteins are synthesized and folded in eukaryotic cells. Likewise, a large amount of viral proteins, including envelope proteins, are synthesized at the ER, which is, therefore, an essential organelle for viral replication and maturation (He, 2006). Indeed, most Flavivirus replicates at a membrane web associated to ER and promote membrane-remodeling events that are driven by hydrophobic transmembrane non-structural viral proteins (Blázquez et al., 2014). Depending on the physiological state and environmental conditions, the protein flux into the ER may vary substantially. In virus-infected cells, the cellular translation machinery is orchestrated by the infecting virus to produce large amounts of viral proteins, which ultimately disturbs ER homeostasis and causes ER stress (Liu et al., 2009; Peña and Harris, 2012; Zhang and Wang, 2012).

Endoplasmic reticulum stress comprises multiple stress response pathways including oxygen and nutrient deprivation, calcium dysregulation, misfolded protein recognition and N-linked glycosylation inhibition (Zhang and Wang, 2012; Sen et al., 2014). Nevertheless, they all converge on the unfolded protein response (UPR). UPR restores the cellular normal function by attenuating protein translation and activating the signaling pathways associated to increased production of molecular chaperones required for protein folding (Zhang and Wang, 2012).

**FIGURE 2:**
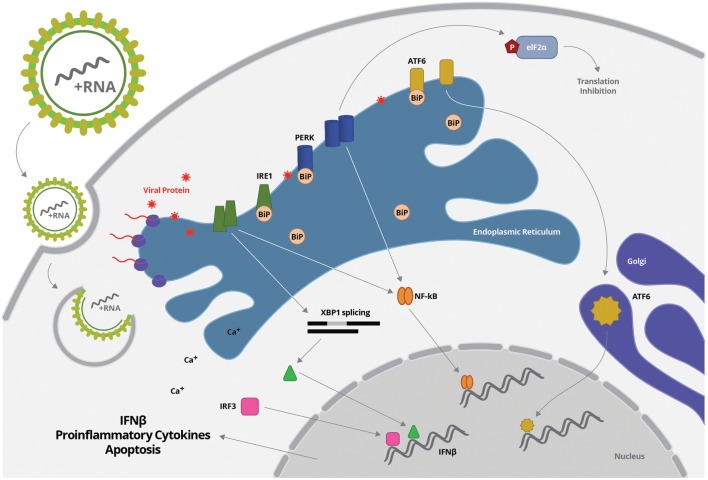
**Flavivirus entry and uncoating is followed by virus RNA translation, which takes place mostly in association to ER membrane.** Increased protein synthesis may disturb ER homeostasis, inducing unfolded protein response, characterized by BiP transiently dissociation and release of PERK, ATF6, and IRE1. BiP releasing from PERK or IRE1 allows the homodimerization of each protein through their luminal domain, which induces their autophosphorylation and subsequent activation. PERK or IRE1 can activated NF-κB. IRE1 also mediated XBP1 splicing, generating functional spliced XBP1 (XBP1s), which is translocated to the nucleus and interact with a conserved site downstream of ifnb1 gene, enhancing IFN- β production. Activation of PERK inhibits protein synthesis through phosphorylation of the eIF2-α. In parallel, the release of BiP from ATF6 promotes the translocation of ATF6 from ER to the Golgi apparatus, where it is cleaved and activated. Activation of ATF6 stimulates the transcription of genes encoding chaperones that refold misfolded proteins.

The UPR consists of three branches of signaling pathways named after the transmembrane ER stress sensors: PKR-like ER protein kinase (PERK), activating transcriptional factor-6 (ATF6), and inositol-requiring protein-1 (IRE1; Garg et al., 2012) (**Figure 2**). BiP is the ER molecule that coordinates all the UPR pathways to restore the cell homeostasis. BiP is a member of heat shock proteins that binds to properly folded and misfolded proteins (Bertolotti et al., 2000). In normal cells, BiP associates with the luminal domains of PERK, ATF6 and IRE1 blocking the activation of UPR pathways (Sou et al., 2012). Under stress conditions, such as Flavivirus infection, BiP transiently associates with viral glycoproteins folding intermediates, releasing PERK, ATF6, and IRE1 to activate UPR pathways (Benali-Furet et al., 2005; Pincus et al., 2010) (**Figure 2**). BiP releasing from PERK or IRE1 allows the homodimerization of each protein through their luminal domain, which induces their autophosphorylation and subsequent activation. Activation of PERK inhibits protein synthesis through phosphorylation of the eIF2-α (Liu et al., 2009; Garg et al., 2012). IRE1 activation leads to the transcription of a subset of genes encoding protein-degradation enzymes (Bertolotti et al., 2000; Pincus et al., 2010). In parallel, the release of BiP from ATF6 promotes the translocation of ATF6 from ER to the Golgi apparatus, where it is cleaved and activated. Activation of ATF6 stimulates the transcription of genes encoding chaperones that refold misfolded proteins (Shen et al., 2005). The three branches of UPR do not operate independently, and the tight temporal control and crosstalk among them constitute an intricate signaling network. Apoptosis is induced when cells are unable to recover from ER stress (Menu et al., 2012).

Endoplasmic reticulum stress-inducing agents synergistically activate type I IFN response (He, 2006; Smith, 2014). In addition, there are increasing evidences that UPR pathway directly enhances cytokine production due to activation of pro-inflammatory transcription factors. Upon ER stress, XBP1 is spliced by IRE1, thereby generating functional spliced XBP1 (XBP1s; Savic et al., 2014) (**Figure 2**). The XBP1 is not only an important component of the UPR pathway, but also an important transcription factor. Spliced XBP1 can then be translocated to the nucleus and interact with a conserved site downstream of *ifnb1* gene, enhancing IFN- β production (Zeng et al., 2010). Virus-triggered UPR pathway also activate IRF3, in the absence of additional stimuli, and this transcription factor binds to the *ifnb1* enhancer sequence, independently of XBP1 (Sato et al., 2000; Sakaguchi et al., 2003; Liu et al., 2012). Little is known about how IRF3 is activated, but the signaling pathway seems to be dependent on calcium release induced by ER stress. Activation of both XBP1 and IRF3 seems to prime the type I IFN response when ER stress is present (Perera-Lecoin et al., 2013) (**Figure 2**).

NF-κB activation is also induced by UPR pathway, leading to the production of proinflammatory cytokines, such as TNF-α and IL-6. Different members of UPR pathway were associated to NF-κB activation, including PERK and IRE-1 (Deng et al., 2004; Tam et al., 2012) (**Figure 2**). In addition, a crosstalk pathway between ER and mitochondrial stress, leading to reactive oxygen species (ROS) accumulation and ER calcium release, was also reported to induce NF-κB activation (Pahl and Baeuerle, 1997). In a positive feedback loop, the resulting inflammatory cytokines can trigger ER stress through induction of further ROS production (oxidative stress) and increased release of calcium from the ER, interfering with chaperone function (Zhang and Kaufman, 2008). Therefore, UPR, or specific pathways within the UPR, can promote inflammatory cytokine production serving as an internal “danger” signal, complementing cellular viral sensors in alerting a cell to invasion and boosting subsequent antiviral immune response (Smith, 2014).

In a sterile inflammation model of ER stress, it was demonstrated that UPR contributes to inflammasome stimulation, promoting IL-1β secretion and cell death (Kim et al., 2013). In this model, activation of IRE1α induced thioredoxin-interacting protein (TXNIP), which activated NLRP3 inflammasome, causing procaspase-1 cleavage and IL-1β secretion (Lerner et al., 2012). In fact, inflammasome-mediated IL-1β secretion after ER stress had been demonstrated in different cell types and it was associated to NF-κB-mediated pro-IL-1β expression, increased ROS production and activation of TXNIP, promoting NLRP3 activation (Kim et al., 2015). Whether UPR pathway, oxidative stress and inflammassome activation crosstalk during flavivirus infection needs further investigation.

It is not surprising that viruses have also evolved mechanisms to counteract UPR pathways to promote their infection. This generally involves regulation of stress response proteins and several molecular chaperones to modulate UPR and increase ER folding capacity that will be addressed below for each specific flavivirus.

#### Flavivirus Infection and ER Stress

Several members of the *Flaviviridae* family including WNV, JEV, DENV, and HCV activate the UPR pathway in a variety of mammalian cells (Su et al., 2002; Tardif et al., 2002; Yu et al., 2006; Medigeshi et al., 2007; Umareddy et al., 2007; Sun W. et al., 2009; Klomporn et al., 2011; Peña and Harris, 2012; Thepparit et al., 2013). The hallmarks of UPR pathway activation observed for Flavivirus was IRE1 mediated splicing of XBP-1, and BiP overexpression in infected cells. By triggering the XBP1 signaling pathway, flaviviruses take advantage of this cellular response, as it is beneficial for viral production and alleviate virus-induced cytotoxicity (Yu et al., 2006).

Regarding DENV infection, it was demonstrated that nonstructural protein NS2B and NS3 are potent inducers of *xbp1* splicing (Yu et al., 2006). Also, in liver cells infected with DENV2, it was observed that PERK and ATF6 were not associated with BiP, resulting in increased eIF-2α phosphorylation (Thepparit et al., 2013).

Japanese Encephalitis Virus infection triggers the UPR pathway in neuronal cells, resulting in apoptotic cell death by robust expression of CHOP/GADD153, a death-related transcription factor that down-regulates Bcl-2 and raises the production of ROS (Liao et al., 2002). ER-mediated UPR induced by JEV also involves stress-inducible p38 mitogen-activated protein kinase (p38 MAPK) activation that could contribute to stimulate CHOP induction at the post-translational level (Su et al., 2002).

West Nile Virus infection also disturbs ER homeostasis leading to activation of all three branches of UPR pathway. Early *xbp1* splicing was detected by 24 h post WNV infection and splicing kinetics corresponded to WNV titers, suggesting that the activation of IRE1 pathway is due to increasing viral load in the ER (Medigeshi et al., 2007). ATF6 cleavage was also detected upon WNV infection, but no change in *atf6* mRNA levels was observed, indicating that this regulation occurs at a posttranscriptional level. WNV infection also leads to eIF-2α phosphorylation by PERK activation and CHOP expression, mediating apoptosis that limited WNV replication (Medigeshi et al., 2007).

The processing and folding of HCV core occurs at the ER and it is strictly dependent on interaction with the ER membrane (Santolini et al., 1994). HCV core expression has been reported to modulate calcium oscillations in T lymphocytes (Bergqvist et al., 2003). In addition, liver cells expressing HCV core showed increased calcium release from ER, which was taken by mitochondria, resulting in high levels of ROS production and decreased antioxidant levels. These events resulted in transitional mitochondria permeability, triggering oxidative stress and apoptosis, which will be better characterized afterward (Choi et al., 2004; Benali-Furet et al., 2005).

Little is known about the role of virus-induced PRR activation on UPR pathway and vice-versa. However, the regulation of RLR, TLR and NLRP3 downstream signaling transduction by ER stress responses had been largely reported in other disease models, such as autoimmune and inflammatory diseases and bacterial infections (Menu et al., 2012; Jiang et al., 2013; Kim et al., 2013; Eckard et al., 2014; Savic et al., 2014). In fact, ER responses were associated to activation of NF-κB, ROS formation and activation of transcription factors that could potentially regulate innate immune responses, as previously described.

Endoplasmic reticulum stress and inflammation might also function in a paracrine way, as observed in other disease models (Garg et al., 2012). It was reported that macrophages cultured with conditioned medium from ER-stressed cells became activated and underwent ER-stress themselves (Mahadevan et al., 2011). Although this mechanism had not been demonstrated for virus infection, this sort of “transmissible stress” could also amplify the inflammatory response and control virus dissemination.

### Mitochondria Stress, ROS Production, and Virus Sensing

**FIGURE 3:**
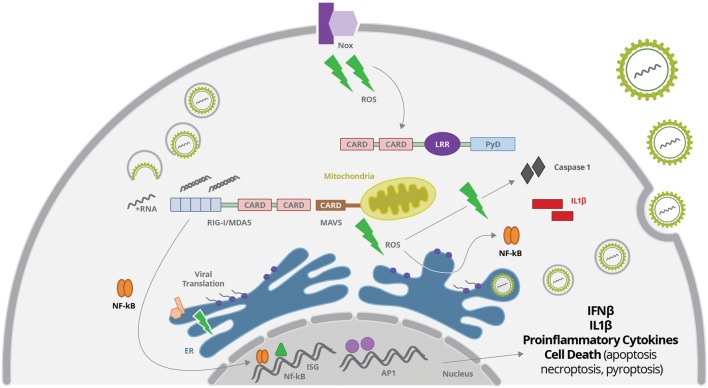
**Virus-derived dsRNA is sensed by cytoplasmic RIG-I, leading to activation of IRFs, NF-κB and AP1 transcription factors, which will promote the secretion of IFNβ and proinflammatory cytokines.** Virus sensing and replication affect mitochondria function leading to reactive oxygen species (ROS) production, what may activated the inflammasome complex, induce oxidative stress with protein oxidation in the ER, and activate NF-κB. ROS may be generated from mitochondrial oxidative phosphorylation, or from activation of membrane NADPH oxidase (Nox), among others not represented here.

Oxidative stress is an event of enhanced formation of so-called ROS in the cell. ROS is a general term indicating a set of molecules and radicals including hydrogen peroxide (H_2_O_2_), superoxide anion (O_2_^-^) and hydroxyl radical (HO; Malhotra and Kaufman, 2007; Reshi et al., 2014). ROS are usually produced during the processes of aerobic metabolism, and ongoing stress, such as exposure to UV light or X-rays, and virus infection (Mittler et al., 2011; Reshi et al., 2014). ROS induces cellular stress through direct interaction with the biological molecules such as proteins, lipids and nucleic acids. One particular ROS species is superoxide (O_2_^-^), which is generated by incomplete electron transfers in the electron transport chain in mitochondria (Paracha et al., 2013). Upon production, O_2_^-^ molecules are rapidly metabolized into hydrogen peroxide (H_2_O_2_). Intermediate concentrations of H_2_O_2_ (and other ROS) result in activation of NF-κB, and activating protein-1 (AP-1), that up-regulate several antioxidant and inflammatory pathways, including ISGs (Schreck et al., 1991, 1992). Many different enzymes at the mitochondria, ER, peroxisomes and other cell compartments are involved in the synthesis of ROS (**Figure 3**). ROS may be generated from mitochondrial oxidative phosphorylation, or from activation of NADPH oxidase, xanthine oxidase, cyclooxygenase, and lipoxygenases, whereas ROS source may differ depending on the initial stimuli (Harijith et al., 2014).

Intracellular scavengers confine ROS production and create a gradient surrounding its source. Therefore, ROS signaling is compartmentalized allowing signal specificity. Disturbing of this compartmentalization by virus replication usually results in increased ROS production, accumulation or efflux, promoting oxidative stress (Reshi et al., 2014). Flavivirus replication in association to ER membranes may result in protein oxidation in ER and production of ROS that results in oxidative stress (Paracha et al., 2013) (**Figure 3**). Other antiviral cellular response, such as autophagy, is also associated with increase of ROS production by accumulation of dysfunctional mitochondria (Nakahira et al., 2011; Scherz-Shouval and Elazar, 2011).

Indeed, viruses frequently modify mitochondrial function through direct interaction of viral proteins with mitochondrial components. Some Flaviviruses changes the steady-state levels of the mitochondrial chaperone prohibitin that disturb the mitochondrial respiratory chain leading to overproduction of ROS (Dang et al., 2011). Also, viral core proteins can bind to the outer mitochondrial membrane, or can be imported into the matrix or to the intermembrane space (Okuda et al., 2002; Ivanov et al., 2013). Viral proteins can also bind to membrane sites closely associated with the mitochondria, such as the mitochondria associated membrane (MAM) fraction of the endoplasmatic reticulum (Chan and Gack, 2015; Vasallo and Gastaminza, 2015). Importantly, Flavivirus dsRNA sensing involving RIG-I activation is directly related to MAVS signal transduction, which requires recruitment of this adaptor to the mitochondrial outer membrane protein (Soucy-Faulkner et al., 2010; Chan and Gack, 2015) (**Figure 3**). Therefore, mitochondrial alteration may directly affect MAVS-stimulated interferon production and inflammatory response.

Nox-derived ROS was reported to be one of the factors related to activation of NF-κB and IRF 3, and subsequent production of IFN-β and IFIT upon RIG-I activation (Soucy-Faulkner et al., 2010; Asdonk et al., 2012; Olagnier et al., 2014b; Kim et al., 2015). Protein sensing by NOD-like receptors (NLRs) was also associated to ROS production induced by Nox2 activation (Lipinski et al., 2009) (**Figure 3**).

The role of oxidative stress in TLR-mediated inflammation was demonstrated in association to TLR4 activation in macrophages and neutrophils, but much less is known regarding virus-mediated activation of TLR3 and TLR7 (Asehnoune et al., 2004; Park et al., 2004). However, it was recently reported that TLR7 activation by RNA virus may enhance Nox activation and oxidative stress, and this may also be true for Flavivirus infection (Lin et al., 2000; Ke and Chen, 2012; Ivanov et al., 2013; Paracha et al., 2013; Olagnier et al., 2014a).

#### Flavivirus Infection and Oxidative Stress

Reactive oxygen species are induced by a number of different Flaviviruses, including HCV, JEV, and Dengue. In many cases, ROS induced by viral infection have been linked with innate antiviral signaling pathways.

Changes in redox status have been associated with increased dengue severity. Adult patients experiencing dengue fever showed altered levels of circulating antioxidants, high levels of biochemical markers of lipid peroxidation (peroxidation potential), and protein oxidation (Malondialdehyde MDA and 4 hydroxyalkenals 4-HDA), as well as increased antioxidant enzymatic activity of superoxide dismutase (SOD; Gil et al., 2004; Klassen et al., 2004). Significant difference was found in protein carbonylation (PCOs) and protein-bound sulphydryl (PBSH) groups in patients with severe dengue. These results suggest a possible role for oxidative stress in plasma proteins and lipid oxidation during DENV-induced pathogenesis (Soundravally et al., 2008; Seet et al., 2009).

Oxidative stress promoted by HCV has been shown to manipulate antioxidant systems, leading to chronic disease (Ke and Chen, 2012; Ivanov et al., 2013; Paracha et al., 2013). Markers of oxidative stress were observed *in vivo* in chronic hepatitis C patients and transgenic mice, as well as in cell lines infected with HCV (Yamaguchi et al., 2005; Ivanov et al., 2013). Almost all HCV proteins, including E1, E2, NS3, NS4B, and NS5A, trigger oxidative stress in HCV infected cells, but HCV core protein is the most potent regulator (Okuda et al., 2002; Thorén et al., 2004; García-Mediavilla et al., 2005; Pal et al., 2010; Ivanov et al., 2011; Ming-Ju et al., 2011). A recent work with human hepatocarcinoma cells (Huh7) showed that oxidative stress caused by HCV core occurs by several independent pathways, such as induction of TGFβ1-dependent expression of NADPH oxidase, upregulation of cytochrome P450 2E1 transcription and expression of ER oxireductin 1α, according to the region of HCV core protein (Ivanov et al., 2015). Moreover, HCV core-induced oxidative stress was also shown to induce RNA damage, leading to enhanced HCV genome heterogeneity and allowing the virus to escape from immune system, as well as from antiviral drugs (Seronello et al., 2011). IL-1β secretion induced by HCV was also dependent on ROS production, in association to activation of NLRP3, ASC and caspase 1, but not on RIG-I sensing, demonstrating that oxidative stress participates in inflammation induced by *Flaviviridae* viruses (Chen et al., 2014).

### Cytoplasmic Stress or Stress Granules

RNA molecules in the cytoplasm can be either directed to active sites of translation represented by polysomes, or may be packed into RNA cytoplasmic granules, called P bodies and SGs, which prevent RNA translation (Anderson and Kedersha, 2006). In fact, when cells undergo environmental stress such as heat shock, UV irradiation, nutrient restriction, hypoxia, ER stress, or viral infection, mRNAs molecules are released from polysomes and the general translation is arrested (Anderson and Kedersha, 2006; Valiente-Echeverría et al., 2012; Reineke and Lloyd, 2013). Different mRNAs in this scenario can be either storage or degraded into SGs and P bodies, which are cytoplasmic granules consisting of RNA-proteins aggregates. P bodies are more dedicated to RNA degradation mechanisms, including RNA association with proteins involved in the RNA induced silence complex (RISC; Beckham and Parker, 2008; Balagopal and Parker, 2009; Lavut and Raveh, 2012). Those mechanisms will not be addressed in this review, since it is also observed under normal physiological conditions. On the other hand, SG were recently described as being part of the cellular response to stress generated by viral infection, especially RNA viruses, such as Flavivirus (Valiente-Echeverría et al., 2012; White and Lloyd, 2012; Onomoto et al., 2014).

**FIGURE 4:**
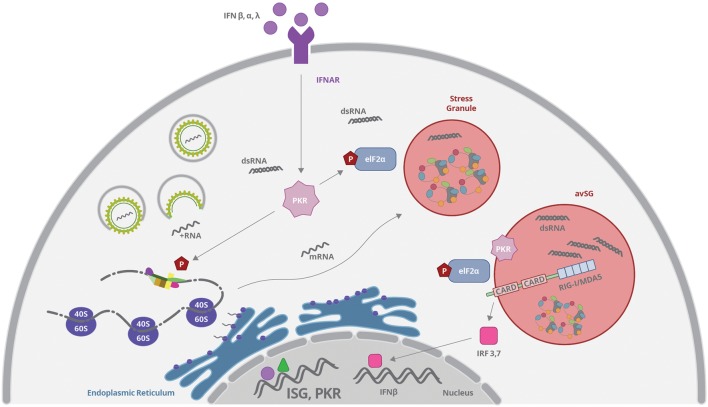
**Viruses can induce stress granules (SGs) formation by interfering with translation complexes or by activating PKR.** PKR expression is upregulated by type I and III IFN binding to IFNAR and the enzyme is activated by dsRNA recognition. Activated PKR phosphorylates eIF2α and promotes mRNP aggregation and SG formation. All these events result in inhibition of protein synthesis. RIG-I, MAVS, and PKR can concentrate and colocalize with SGs induced by virus infection, forming the so-called antiviral stress granules (avSG), which may function as a platform for RNA sensing and IFN response stimulation.

The critical event to promote SG formation is the phosphorylation of eIF2, preventing the assembly of the active ternary preinitiation complex eIF2-GTP-tRNAMet, which results in polysome disassembly and blockade of translation initiation (Kedersha et al., 2002; Weber et al., 2008; Valiente-Echeverría et al., 2014) (**Figure 4**). As mentioned earlier, eIF2 phosphorylation can be mediated by a family of serine/threonine kinases including HRI, PKR, PERK/PEK, and GCN2 (Kedersha et al., 2005; Mazroui et al., 2006). The intracellular localization of each kinase is associated to the different pathways and stress sensors that culminate in the final event of eIF2 phosphorylation and translation inhibition. The HRI kinase (eIF2K1) is activated in heme deprivation and oxidative stress with ROS release; PKR is the cytoplasmic kinase (eIF2K2) activated by viral infection and foreign dsRNA; PERK/PEK (eIF2K3) is associated to ER and related to UPR pathway, as described before; and GCN2 (eIF2K4) is activated by amino acid starvation and UV irradiation (Bertolotti et al., 2000; Daito et al., 2014; Jain et al., 2014). Those kinases cause the phosphorylation of the eIF2-α at Ser51, which impairs its binding to eIF2B, inhibiting GDP-GTP exchange, which is necessary for ^Met^tRNA position on the 43S ribosomal subunit and initiation of translation (Kedersha et al., 2002). Other mechanisms independent of the phosphorylation of eIF2 have also been described (Mazroui et al., 2006; White and Lloyd, 2012).

Stress granules are very dynamic sites and are formed from condensation of stalled translation initiation complexes including 43S and 48S ribosomal preinitiation complexes (Kedersha et al., 2005). However, these translation complexes can be rapidly released to resume protein synthesis when stress conditions are ceased. The molecular mechanism by which SG condense is still in debate and involves the self-oligomerization of key constituent RNA-binding proteins, such as Ras-Gap SH3-binding protein (G3BP1), T-cell restricted intracellular antigen 1 (TIA-1) and TIA-1-related protein (TIAR; Kedersha et al., 1999; Piotrowska et al., 2010; Valiente-Echeverría et al., 2014). Moreover, hundreds of RNA-interacting proteins and an siRNA were described to be located into SG including: translation initiation factors (eIF4E, eIF4G, eIF4A, eIF4B, eIF3, and eIF2), poly(A)-binding protein (PABP1) and others RNA binding proteins that regulate mRNA structure and function(Kedersha et al., 2005; Anderson and Kedersha, 2009). These RNA binding proteins include: human antigen R (HuR); dsRNA-binding protein (Staufen1); polysomal ribonuclease 1 (PMR-1); the posttranscriptional regulator Smaug (SAMD4A); tristetraprolin (TTP); T-cell restricted Fragile × Mental Retardation Protein (FXMR/FXR1); RNA helicase (RCK/p54/DDX6); cell cycle associated protein 1 (caprin1); HuR; Y-box binding protein 1 (YB-1), that activates the 5′ UTR of G3BP1 mRNAs and cytoplasmic polyadenylation binding protein (CPEB; Anderson and Kedersha, 2006).

The composition of SGs can vary depending on the type of cell stress, however, stalled translation complexes are common components of these RNA granules. SGs induced by virus infection are enriched in G3BP1 and RNA binding protein Sam68, which is part of signaling transduction pathways during alternative splicing, RNA-3′UTR formation and cell cycle regulation (Piotrowska et al., 2010; Finnen et al., 2012; Valiente-Echeverría et al., 2014).

#### SG Formation and RNA Virus Sensing

Viruses can induce SGs formation by interfering with translation complexes such as eIF4G or eIF4A (White and Lloyd, 2012). However, induction of SG upon virus infection is more commonly associated to activation of PKR by recognition of viral dsRNA (**Figure 4**). Activated PKR phosphorylates eIF2α, as described before, and promotes mRNP aggregation induced by G3BP protein (Tsai and Lloyd, 2014). Interestingly, it has been recently demonstrated that cytoplasmic virus sensor and interferon responsive genes such as RIG-I, MDA5, MAVS, and PKR can concentrate and colocalize with SGs induced by virus infection, forming the so-called antiviral stress granules (avSG; Onomoto et al., 2012; Langereis et al., 2013) (**Figure 4**). Some authors propose that avSG may function as a platform for RNA sensing and IFN response stimulation (Onomoto et al., 2012; Yoo et al., 2014). On the other hand, inhibition of protein synthesis induced after SG formation may diminish the translation of IFN-induced genes as a viral strategy to prevent immune response (Onomoto et al., 2014). Different groups have been investigating these events and, although some of them have been using surrogate dsRNA agents (like poli I:C), it is plausible to assume that transient avSG formation may be a common phenomenon upon virus infection, including Flaviviruses.

RIG-I translocation to SG containing PKR, OAS, and RNAse L was demonstrated upon viral infection or viral RNA transfection (Chakrabarti et al., 2012). In this model, PKR activation was essential for the formation of RIG-I containing SG, and was necessary for IRF3 dimerization and viral-mediated IFN response (**Figure 4**). PKR activation was completely dependent on the presence of virus RNA (Gilfoy and Mason, 2007). The complete signaling pathway involved in the formation and response of avSG is not fully understood, although it was recently reported that dsRNA-activated MAVS can directly interact with PKR, through CARD domain, promoting PKR activation, and subsequently eIF2-α phosphorylation followed by SG assembly (Zhang et al., 2014). Silencing of MAVS or PKR limited SG formation induced by dsRNA, and it was proposed that association of MAVS with PKR might directly affect the dsRNA-dependent dimerization of the latter. However, the role of RIG-I and MDA5 was not as important as MAVS in the system, suggesting the involvement of additional undefined pathways.

Although PKR had been demonstrated to be a key element for the generation of avSG as a platform that allows interaction between antiviral proteins and non-self RNA ligands, it might have different roles, depending on the stimulated cell type (Onomoto et al., 2012; Yoo et al., 2014). PKR silencing resulted in decreased IFN-β production in hepatoma cells infected with DENV, but not in fibroblast nor macrophages (Li Y. et al., 2013). In addition, decreased protein synthesis induced by PKR activation might, in fact, inhibit the synthesis of ISG proteins, in spite of increased mRNA expression triggered by the RNA sensor (Garaigorta and Chisari, 2009).

#### Modulation of SG Assembly by Flavivirus

Since viral propagation completely depends on the host translational machinery, induction of SG by virus infection is usually transitory and most viruses suppress SG assembly at some point of their replicative cycle. In general, flaviviruses such as WNV, DENV, and JEV block arsenite-induced stress granule formation by hijacking multiple SG components, including TIA1, TIAR, G3BP1, and Caprin-1 (Onomoto et al., 2014).

Li et al. (2002) first demonstrated that SG proteins directly interact with WNV RNA and proteins (Li et al., 2002). They observed that TIA-1 and, mostly, TIAR interacts with minus strand 3′ terminal stem loop RNA (3′ SL RNA) of WNV, which is the initiation site of genomic RNA synthesis (Li et al., 2002). Interaction of virus RNA with TIA-1/TIAR seemed to facilitate WNV replication and, TIAR knockout cells exhibited decreased WNV replication when compared with control cells (Li et al., 2002). DENV RNA also binds specifically to TIA-1, TIAR, and G3BP (Emara and Brinton, 2007; Bidet et al., 2014). In addition, a recent study showed that caprin-1 interacts with the DENV 3′UTR suggesting that this genomic region is a site for assembly of SG proteins (Ward et al., 2011). Both flavivirus, DENV and WNV, inhibited SG formation and eIF2-α phosphorylation, preventing the shutoff of host proteins translation, and favoring virus RNA translation (Emara and Brinton, 2007). It is likely, therefore, that recruiting SG proteins to different compartments may allow virus translation or RNA replication and prevents innate immune response as a consequence of SG assembly.

Japanese Encephalitis Virus core protein also recruited several SG-associated proteins, including G3BP and USP10, in a way dependent on caprin-1 binding (Katoh et al., 2012). These interactions were associated to the suppression of SG assembly, resulting in increasing viral replication. SGs enriched with G3BP1/eIF3/eIF4B were also detected upon TBEV infection. Depletion of TIA-1 or TIAR resulted in increased production of new infectious virus, indicating that SGs harboring TIAR and TIA-1 inhibit TBEV replication (Albornoz et al., 2014).

Hepatitis C Virus also exploits SG machinery by recruiting PKR-eIF2-α phosphorylation pathway as a strategy for viral escape (Garaigorta and Chisari, 2009; Garaigorta et al., 2012). It has been reported that IFN treatment of HCV-infected cells induced phosphorylation of PKR and eIF2-α, thereby inhibiting *de novo* cellular protein synthesis, including translation of antiviral interferon-stimulated genes. Indeed, IFN-stimulated proteins like MxA and USP18 levels were inversely correlated with the amount of SGs in HCV infected Huh7 cells, suggesting that interferon-stimulated gene translation was inhibited in SG-containing infected cells (Garaigorta et al., 2012). Activation of PKR, however, did not inhibit translation of HCV proteins, probably due to IRES-dependent synthesis (Garaigorta and Chisari, 2009).

Early and late stages of HCV replication seems to be dependent on SG components. Short hairpin RNA (shRNA) knockdown experiments suggested that TIA-1, TIAR, and G3BP1 were required for efficient HCV RNA and protein accumulation at early time points after HCV infection (Garaigorta et al., 2012). In addition, G3BP1, ATX2, PABP1, and USP10 colocalized with HCV core protein at lipids droplets, suggesting that they might facilitate RNA packaging and virus assembly (Ariumi et al., 2011; Pager et al., 2013). G3BP1 was showed to interact with the 5′ end of the HCV minus-strand RNA and with NS5B protein suggesting that G3BP1 is required for HCV replication. Indeed, RNAi mediated depletion of SG components decreased the expression of HCV core and NS5A proteins, and inhibited assembly and release of HCV virions (Pager et al., 2013).

In this sense, the subversion of another SG-associated pathway has been recently unveiled and involves the ubiquitous ATP-dependent RNA helicase DDX3X, which is a pivotal cell factor for HCV replication and SG assembly (Li Q. et al., 2013; Pène et al., 2015). Interaction of DDX3X with HCV 3′ UTR had been previously shown to induce IKK-α activation and cellular lipogenesis, which benefited virus assembly (Li Q. et al., 2013). It was then demonstrated that DDX3X recognition by HCV 3′UTR and IKK-α activation, promoted its redistribution to G3PB-containing SG and posterior association with HCV core protein at lipid droplets (Pène et al., 2015). Knockdown of DDX3X and multiple SG components inhibited HCV infection, suggesting that dynamic associations of DDX3X, HCV RNA and host proteins at lipid droplets surfaces might play a crucial role in HCV replication (Pène et al., 2015).

Ruggieri et al. (2012) showed that HCV exhibited a stress response oscillation as a mechanism to prevent long-lasting translation repression (Ruggieri et al., 2012). Changes in active and repressed phases of RNA translation were observed during HCV infection of IFN-α treated Huh7 liver cells, inducing highly dynamic assembly/disassembly of cytoplasmic SGs. In this case, eIF2-α phosphorylation was counteracted by GADD34 upregulation, a regulatory subunit of protein phosphatase 1 that reverted the phosphorylation of eIF2-α, reactivating translation (Ruggieri et al., 2012).

### Inflammasome Activation upon Virus-Induced Stress

Virus sensing and the resulting metabolites available upon cell stress may be intimately associated to the activation of inflammasome complexes, which are a signaling platforms, containing a PAMP/DAMP sensor from NLR or PIHYN families, an adaptor protein containing CARD domain (ASC) and caspase 1 (Henao-Mejia et al., 2012). Stimulation of the inflammasome ultimately leads to activation of caspase 1/11, leading to IL-1β and IL-18 secretion and, sometimes pyroptosis cell death (Poeck et al., 2010). Inflammasome activation may be triggered by different signals, including infection, altered concentration of secondary metabolites, or ion influx (**Figures 1** and **3**). A first signal, usually triggered by other innate immune receptors such as TLR or RIG-I, induces the production of immature pro-IL-1β and pro-IL-18. A second signal, then, leads to caspase 1/11 activation, which cleaves pro-IL-1β and pro-IL-18, generating mature cytokines, which may then be secreted (Lupfer et al., 2015; Vanaja et al., 2015).

NLRP3 is the best-known NLR associated to inflammasome activation. Although the whole mechanism associated to NLRP3 activation is still not fully understood, it is well known that, once activated, it associates to ASC and procaspase 1, promoting its activation (Motani et al., 2011; Zhou et al., 2011). NLRP3 is activated by several stimuli, such as ATP, uric acid and cholesterol crystal, among others. Several NLRP3 activators are associated with ROS production, which, in turn may be either important for NLRP3 activation or expression (Bauernfeind et al., 2011; Rahman and McFadden, 2011; Zhou et al., 2011). In fact, NLRP3 inflammasome is very sensitive to ROS and most signals leading to activation of NLRP3 inflammasome, including virus infection, are ROS-dependent (Bauernfeind et al., 2011) (**Figures 1** and **3**). In addition, cell death and release of cellular content, such as ATP may also promote inflammasome activation in bystander cells.

NLRP3 activation may take place at the mitochondria and MAVS had been reported as a possible adaptor protein associated to NLRP3 recruitment to this organelle (Zhou et al., 2011; Subramanian et al., 2013). In this case, MAVS participation might not depend on RIG-I activation. In fact, MAVS association to RIG-I or NLRP3 were reported to be mutually exclusive in some systems (Subramanian et al., 2013). MAVS interaction with either RIG-I or NLRP3 could be dictated by the expression levels of each sensor, or by the available PAMP, and both elements might be determinant to the subsequent triggered signaling pathway, resulting in either IFN-β or IL-1β production. RIG-I might also directly activate inflammasomes, in a MAVS-independent way (Poeck et al., 2010). Alternatively, RLR and inflammasome might be sequentially stimulated. In epithelial infection with influenza virus, it was demonstrated that RIG-I was the primary viral RNA sensor, inducing IFN-β secretion (Loo et al., 2008; Pothlichet et al., 2013). RIG-I-induced IFN then up-regulated TLR3 and NLRP3 and all these mediators (RIG-I, TLR3, NLRP3, and IFN) were necessary for caspase 1 activation and IL-1β release (Pothlichet et al., 2013).

Caspase 1 has more than a hundred substrates, indicating that inflammasome may function far beyond the inflammatory response (Henao-Mejia et al., 2012). Cell death is one possibility downstream inflammasome activation, and this event may be a complex process involving cross modulation of adaptor proteins associated to different death mechanisms. In most cases, inflammasome-dependent caspase 1 activation leads to pyroptosis, which may contribute to the amplification of the inflammatory response by promoting the release of intracellular content (Bergsbaken et al., 2009; Labbé and Saleh, 2011; Miao et al., 2011).

Hepatitis C Virus may activate different virus sensing and inflammation pathways, depending on the cell type. TLR3 showed to be essential for type I IFN production in hepatocytes upon HCV infection, whereas TLR7 was essential for this response in pDC (Chattergoon et al., 2014). On the other hand, in monocytes, activation of TLR7 by HCV virions was associated to activation of the inflammasome pathway, promoting the secretion of IL-1β and IL-18 in a NLRP3-dependent and infection independent pathway (Chattergoon et al., 2014). Other authors could not observe activation of inflammasome by HCV virions, but also found that HCV RNA was able to directly stimulate these platforms dependent on NLRP3, ASC, caspase 1 and ROS (Chen et al., 2014).

Activation of NLRP3 inflammasomes was also observed upon dengue infection of macrophages, promoting the secretion of IL-1β and pyroptosis (Wu et al., 2013). Interestingly, it was recently demonstrated that inflammasome activation might also take place in platelets, and was induced by dengue infection (Hottz et al., 2013). Activation of NLRP3 and increased ROS production promoted IL-1β release from platelets, which were then able to increase vascular permeability. Taken together, these data suggested that inflammasome activation by dengue might be a key mechanism for disease pathogenesis, and might be associated to altered platelet and vascular function, cell death of immune cells, and fever (Hottz et al., 2013).

Increased IL-1β and activation of NLRP3 inflammasomes were also detected during infection of flaviviruses associated to CNS, including JEV and WNV (Kaushik et al., 2012; Kumar et al., 2013). In a mouse experimental model of WNV infection, IL-1β secretion was associated to inhibition of virus replication in neurons, increased production of IFN-β, modulation of pro-inflammatory cytokines, and protection from neuronal cell death and tissue damage in CNS (Ramos et al., 2012; Kumar et al., 2013).

### Virus Infection Inducing Stress and Death

Prolonged stress induced by any of the mechanism described above may result in cell death. For example, ablation of RIG-I or MAVS in a number of virus infection models resulted in increased cell survival, indicating a direct role of dsRNA sensing for the induction of cell death (Chattopadhyay et al., 2010; Yu et al., 2010). In fact, RIG-I activation stimulates several apoptosis-related genes, either by a direct effect of IRF3 activation or through secondary IFN-mediated responses, which can also amplify the response induced by IRF. RIG-I activation by HCV, for example, promoted TRAIL-mediated apoptosis, although it was not clear whether this was a direct effect of RIG signaling or a secondary, IFN-mediated effect (Yang et al., 2011). Other studies reported that IRF-3 translocation to the nucleus directly stimulated the expression of ISG, such as IFIT1 and IFIT2 (ISG54 and ISG56), which activation was previously associated to caspase3-dependent, mitochondrial associated apoptosis (Stawowczyk et al., 2011).

It was also proposed that IRF3-induced apoptosis might happen independently of its transcription factor activity. In this sense, IRF3 activation by cytoplasmic dsRNA-RIG-I complex induced its association to proapoptotic Bax, leading to the translocation of both proteins to the mitochondria and induction of Bax-mediated, caspase 9-dependent apoptosis (Chattopadhyay et al., 2010). According to this model, activated IRF3 may either translocate to the nucleus or to the mitochondria, but the mechanisms regulating both pathways are not completely understood.

Interestingly, it had been showed that caspase activation induced by virus infection resulted in MAVS cleavage and consequent inhibition of interferon production, but did not result in increased virus titers (Yu et al., 2010). Therefore, caspase activation upon RIG-I stimulation may help to control the inflammatory response, suggesting a close relation between virus sensing, stress signals and inflammation.

Activation of RLR/MAVS pathway may also result in necroptic cell death. Stimulation of pDC with poli:C, a dsRNA surrogate, induced the release of cathepsin D from the lysosomes to the cytoplasm, where it associated to MAVS, and was recruited to a complex formed by MAVS-caspase 8-RIPK1 in the mitochondria. Cathepsin D cleaved caspase 8, which probably promoted its release from mitochondria, allowing activation of RIPK and initiation of necroptosis (Zou et al., 2013). The consequence of necrosis is the release of several cellular constituents, such as nucleic acids and ATP, which may then stimulate other receptors expressed in bystander cells, like inflammasome-related sensors.

Finally, other cell mediators produced after virus sensing may also modulate survival of infected and bystander cells. Engagement of TNFR by TNF-α can result in cell survival or promote apoptosis or necrosis, which will depend on NF-κB activation, or on the balance between RIPK1 and caspase 8 activation. Therefore, the other signals triggered by virus infection in association to TNFR-driven signaling will then dictate the cell fate.

Importantly, apoptotic or necrotic death is not always the cellular fate upon virus infection, due to rescue mechanisms, such as inhibition of protein synthesis and autophagy. For instance, virus-induced ROS accumulation stimulates RLR- and NLRP-mediated responses. However, these events may also stimulate the autophagy machinery, which will then limit ROS and mitochondria signals and restrain PRR downstream responses. A crosstalk between cellular rescue mechanisms and PRR-induced downstream signaling is, then, important to control virus replication while maintaining tissue homeostasis.

## Conclusion

**FIGURE 5:**
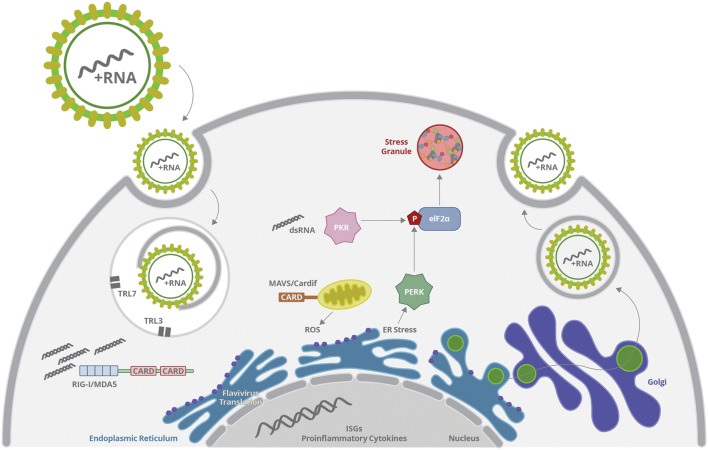
**Flavivirus infection is initiated by virus endocytosis, envelope fusion and genome uncoating, releasing virus RNA.** Genomic RNA or dsRNA generated during replication may be sensed by vesicular TLR, RIGI, MDA5, or PKR. Activation of TLR and RIG-I promotes ISG and inflammatory genes expression; whereas PKR stimulation promotes eIF-2α phosphorylation and stress granules formation. In parallel, virus protein translation takes part mostly at the ER, altering ER homeostasis and promoting UPR. Virus sensing and replication affect mitochondrial function, inducing increased ROS production and oxidative stress, what may activate other inflammatory signal and affect cell survival. Virus scape mechanisms are triggered at different levels in different cell types, allowing some level of progeny release.

The RNA genome and proteins of *Flaviviridae* members can be sensed by several cellular mechanisms, which will induce immune responses and activate cellular stress pathways, including include ER stress, oxidative stress and stress granules formation, restricting virus production (**Figure 5**). However, viruses develop several mechanisms to subvert the innate antiviral responses by sequestering cellular partners related to inflammation and stress in order to delay protein translation inhibition, inhibit interferon responses and cell death. Virus scape mechanisms may be triggered at different levels in different cell types, allowing some level of progeny release.

The interplay between innate immune response and cellular stress pathways is just beginning to be elucidated and several key questions remains unclear. *Flaviviridae* members infect different cell types, and the virus sensing molecules and the persistence of cellular stress will depend directly on virus intracellular trafficking through the cell compartments. Thus, it is not only important to follow multiple components of inflammation and cellular stress during infection, but also to examine the functional consequences of these cellular responses to the disease pathogenesis. Some Flavivirus described here are related to aggressive pathologies such as Hemorrhagic Fever and neurological disorders. In addition, the biology and pathogenesis of other Flavivirus, such as ZIKV is largely unknown. The balance between stress, inflammation and antiviral responses will determine a successful control of virus dissemination or the development of severe disease. Since stress responses and innate immunity likely crosstalk at multiple levels, these pathways may be exploited as a broad spectrum antiviral strategy.

## Author Contributions

AV, RA, and LA wrote the review. RA and LA critically revised the review. LA was responsible for conception and design of the review.

## Conflict of Interest Statement

The authors declare that the research was conducted in the absence of any commercial or financial relationships that could be construed as a potential conflict of interest.
